# Severe Acute Kidney Injury with Necrotizing Glomerulonephritis After Piperacillin/Tazobactam Therapy in a Patient with Peritonitis: A Case Report and Literature Review

**DOI:** 10.3390/diagnostics15050574

**Published:** 2025-02-27

**Authors:** Youn-Sik Oh, Man-Hoon Han, Yong-Jin Kim, You Hyun Jeon, Hee-Yeon Jung, Ji-Young Choi, Jang-Hee Cho, Sun-Hee Park, Chan-Duck Kim, Yong-Lim Kim, Jeong-Hoon Lim

**Affiliations:** 1 Department of Internal Medicine, School of Medicine, Kyungpook National University, Kyungpook National University Hospital, Daegu 41944, Republic of Korea; aticucu@naver.com (Y.-S.O.); yh-jeon@knu.ac.kr (Y.H.J.); hy-jung@knu.ac.kr (H.-Y.J.); jyss1002@hanmail.net (J.-Y.C.); jh-cho@knu.ac.kr (J.-H.C.); sh-park@knu.ac.kr (S.-H.P.); drcdkim@knu.ac.kr (C.-D.K.); ylkim@knu.ac.kr (Y.-L.K.); 2 Department of Pathology, School of Medicine, Kyungpook National University, Kyungpook National University Hospital, Daegu 41944, Republic of Korea; mhhan1@knu.ac.kr (M.-H.H.); yyjjkim@knu.ac.kr (Y.-J.K.)

**Keywords:** piperacillin/tazobactam-induced nephrotoxicity, acute kidney injury, drug-induced acute interstitial nephritis, necrotizing glomerulonephritis, antibiotic-related nephrotoxicity

## Abstract

Piperacillin/tazobactam (PT), a widely utilized broad-spectrum antibiotic, has been associated with acute kidney injury (AKI). Although the precise mechanism remains uncertain, and most cases of PT-associated AKI are mild, this report describes a rare and severe complication of PT, which manifested as severe AKI with necrotizing glomerulonephritis requiring hemodialysis. A 42-year-old man was transferred to the nephrology clinic due to progressive deterioration of kidney function. Prior to the transfer, the patient had been diagnosed with appendicitis complicated by peritonitis and received intravenous PT for 8 days. Baseline kidney function was normal, but serum creatinine subsequently increased to 7.2 mg/dL. Hemodialysis was initiated to address metabolic acidosis and edema. Kidney biopsy revealed severe acute tubular injury and necrotizing glomerulonephritis. Steroid therapy was initiated based on the biopsy findings, and serum creatinine returned to normal levels after 4 weeks of treatment. This case demonstrates that severe AKI with necrotizing glomerulonephritis can occur after PT use. Prompt kidney biopsy and the timely initiation of immunosuppressive therapy are essential for a favorable outcome.

## 1. Introduction

Acute kidney injury (AKI) is a condition of sudden and clinically significant decline in kidney function and is defined by at least one of the following criteria: an increase in serum creatinine by ≥0.3 mg/dL (≥26.5 µmol/L) within 48 h, an increase in serum creatinine to ≥1.5 times the baseline level within the prior seven days, or a reduction in urine output to <0.5 mL/kg/h for at least six hours [1]. AKI is associated with an increased risk of adverse outcomes, including morbidity and mortality [2,3,4]. Among the various etiologies of AKI, drug-induced nephrotoxicity is a major contributor, responsible for up to 60% of cases. Antibiotics are an important cause of drug-induced AKI; many commonly used agents in clinical practice are capable of precipitating its development [5]. Given the widespread use of antibiotics for various infectious diseases, understanding their nephrotoxic potential is important for preventing AKI and mitigating its complications.

Piperacillin/tazobactam (PT) is a combination antibiotic consisting of an antipseudomonal penicillin and a beta-lactamase inhibitor. Due to its broad-spectrum coverage, PT is widely utilized in the treatment of nosocomial and healthcare-associated infections, including intra-abdominal infections, pneumonia, complicated urinary tract infections, sepsis, and febrile neutropenia [6,7]. PT is often preferred due to its efficacy against a wide range of Gram-positive, Gram-negative, and anaerobic bacteria. Although PT is generally considered safe, several observational studies have documented an increased risk of AKI after its use [6,8,9].

Post-marketing surveillance in Japan revealed an AKI incidence of 0.4% (2 out of 486) among a general population of patients treated with PT [6]. Whereas, a markedly higher incidence of 18.2% was observed in a study focused on older Japanese patients [8], suggesting that age and underlying co-morbidities may influence susceptibility to PT-induced nephrotoxicity. A study in the United States identified AKI in 11.4% of patients receiving PT [10]. They also reported that the addition of vancomycin to PT increased the risk of AKI by 1.7-fold compared to PT alone. Another large retrospective cohort study of critically ill patients reported that the use of PT was associated with a higher incidence of AKI and also an increased risk of renal replacement therapy compared with alternative beta-lactam antibiotics [11]. These findings highlight the variability in reported AKI incidence associated with PT use and suggest the need for further studies of its pathophysiologic mechanism, particularly in patients with infectious diseases.

The exact pathogenesis of PT-associated AKI remains unclear. However, evidence from case reports and experimental studies suggests that immune-mediated mechanisms and direct tubular toxicity are involved [5,12]. Some reports have proposed a mechanism of acute interstitial nephritis (AIN) in which hypersensitivity reactions lead to inflammatory infiltration of the renal tubules, resulting in AKI. In such cases, eosinophiluria, pyuria, and elevated inflammatory markers may be observed [5]. Other studies have suggested that PT may cause AKI via direct tubular toxicity, leading to mitochondrial dysfunction and oxidative stress within renal tubular cells [12].

Given the potential for AKI associated with the use of PT, careful patient selection and monitoring are required to minimize the risk of nephrotoxicity. Risk factors such as advanced age, pre-existing kidney disease, concomitant use of other nephrotoxic drugs (e.g., vancomycin, aminoglycosides, non-steroidal anti-inflammatory drugs, and contrast agents), and sepsis should be considered before initiating PT therapy. Regular monitoring of serum creatinine, estimated glomerular filtration rate, and urine output is essential for early detection of renal dysfunction.

This case report describes a severe instance of AKI with necrotizing glomerulonephritis after PT administration in a previously healthy young patient. This case highlights the importance of recognizing PT as a potential nephrotoxic agent and emphasizes the need for close monitoring of kidney function during PT therapy.

## 2. Detailed Case Description

**Figure 1:**
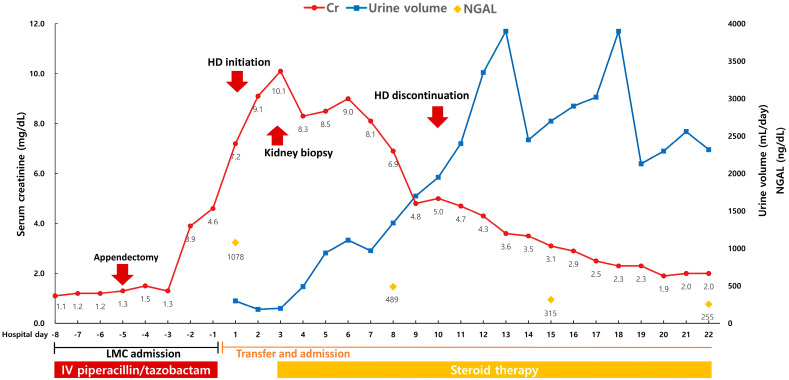
Overview of the clinical course. Abbreviations: Cr, serum creatinine; HD, hemodialysis; IV, intravenous; LMC, local medical center; NGAL, neutrophil gelatinase-associated lipocalin.

In June 2024, a 42-year-old man without an underlying disease was diagnosed with appendicitis complicated by peritonitis. The patient complained of fever, poor oral intake, and generalized malaise for several days. He also reported progressive abdominal discomfort, which had intensified over the past 48 h. On physical examination, the patient appeared acutely ill, with notable tenderness in the lower abdomen, particularly in the right lower quadrant. The initial serum creatinine level was 1.1 mg/dL. Abdominal computed tomography (CT) confirmed the presence of acute appendicitis with evidence of perforation and secondary peritonitis. The patient was receiving empiric antibiotic therapy for a complicated intra-abdominal infection and was scheduled to receive 7 days of PT. He received 4.5 g of PT intravenously every 8 h. Over the next 5 days, his fever subsided, and inflammatory markers showed a declining trend, indicating an improvement in the acute infectious process. Subsequently, laparoscopic appendectomy was performed without intraoperative complications, at which time the creatinine level was 1.3 mg/dL. The post-operative course was stable without complications; however, on post-operative day 3, his serum creatinine levels began to rise to 3.9 mg/dL despite adequate hydration and the absence of evert nephrotoxic exposures. By the eighth day of PT therapy, the serum creatinine level had markedly increased to 7.2 mg/dL, and urine output had declined to less than 500 mL/day, consistent with severe AKI (Figure 1). The patient was subsequently transferred to the nephrology department of a tertiary hospital for further evaluation and management.

At admission to the nephrology unit, the patient was hemodynamically stable with a blood pressure of 147/79 mmHg, a heart rate of 64 beats per minute, a respiratory rate of 16 breaths per minute, and the body temperature was 37.3 °C. His height was 175 cm, weight was 73 kg, and a body mass index was 23.8 kg/m^2^. Physical examination revealed generalized edema involving the lower extremities, peri-orbital region, and mild ascites. Pulmonary rales were also heard. The patient had no history of chronic illnesses such as diabetes mellitus or hypertension and had no prior episodes of kidney disease. He also denied the use of nephrotoxic agents, including non-steroidal anti-inflammatory drugs or herbal supplements. His medication history was limited to the empirical antibiotic therapy initiated for his appendicitis. Abdominal CT showed no residual signs of intra-abdominal infection or peritoneal abscess, but mild ascites and bilateral pleural effusions were noted. The kidneys appeared normal in size and shape with no signs of hydronephrosis or obstruction.

Laboratory analysis at the time of transfer revealed increased inflammatory markers; white blood cell count was elevated at 25.9 × 10^9^/L, and C-reactive protein level was high at 16.5 mg/dL. Markers of renal function markers were also elevated; blood urea nitrogen was significantly elevated at 79.8 mg/dL, serum creatinine level was elevated to 7.2 mg/dL, and serum cystatin-C level was elevated at 4.96 mg/L. Neutrophil gelatinase-associated lipocalin (NGAL), a marker of tubular injury and AKI, was significantly elevated at 1078.5 ng/mL (normal: <150 ng/mL). Serum electrolyte levels were as follows: sodium, 131 mmol/L; potassium, 4.0 mmol/L; calcium, 7.7 mg/dL; phosphate, 2.9 mg/dL; and uric acid, 13.7 mg/dL. Serum protein and albumin levels were 5.1 g/dL and 2.8 g/dL, respectively. Venous blood gas analysis showed a pH of 7.3, a pCO_2_ of 23.6 mmHg, and a bicarbonate level of 15.6 mmol/L, indicating mild metabolic acidosis compensated by respiratory alkalosis. Urine output was reduced to 300 mL/day; urinalysis detected microscopic hematuria (3+) and proteinuria (4+), with a spot urine protein-to-creatinine ratio of 22.0 g/g creatinine. Immunologic tests for glomerulonephritis, including anti-nuclear antibodies, anti-neutrophil cytoplasmic antibodies, anti-myeloperoxidase and anti-proteinase 3 antibodies, anti-glomerular basement membrane antibodies, and anti-phospholipase A2 receptor antibodies, complement C3 and C4 levels, and viral markers for hepatitis B, hepatitis C, and human immunodeficiency virus were negative.

**Figure 2:**
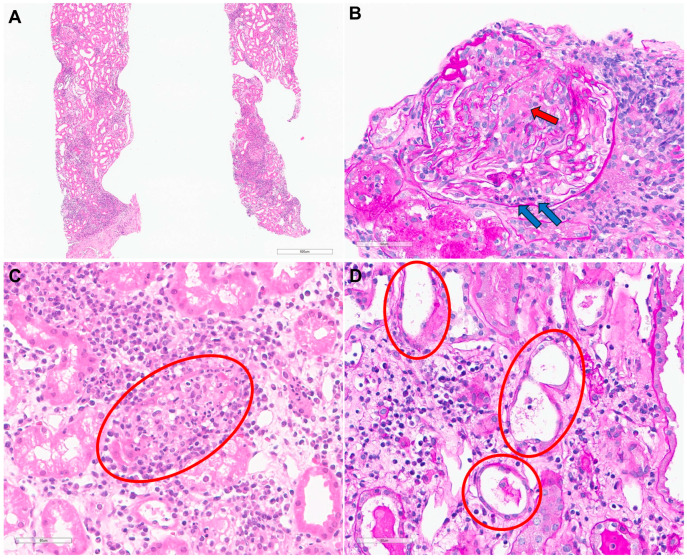
Histopathological findings of kidney biopsy. (**A**) Moderate mixed inflammatory cell infiltration in the interstitium (hematoxylin and eosin; original magnification ×40). (**B**) Necrotizing glomerulonephritis with fibrinoid necrosis (red arrow) and neutrophil infiltration (blue arrows) (periodic acid-Schiff; original magnification ×400). (**C**) Severe interstitial inflammation with neutrophil infiltration in the tubular epithelium (circle) (hematoxylin and eosin; original magnification ×400). (**D**) Acute tubular injury characterized by tubular epithelial flattening and loss of the brush border (circles) (periodic acid-Schiff; original magnification ×400).

Considering the rapid deterioration in kidney function accompanied by edema, hemodialysis was immediately initiated to manage uremic symptoms and fluid overload. A kidney biopsy was performed on hospital day 3 to further elucidate the etiology of the AKI. Light microscopy revealed moderate mixed inflammatory cell infiltration within the interstitium (Figure 2A). Periodic acid-Schiff staining identified necrotizing glomerulonephritis with fibrinoid necrosis and neutrophil infiltration (Figure 2B), as well as severe interstitial inflammation with neutrophil infiltration in the tubular epithelium (Figure 2C). Findings consistent with acute tubular injury, including flattening of the tubular epithelium, loss of the brush border, and intra-tubular debris were also observed (Figure 2D). Immunofluorescence staining showed no significant staining, and electron microscopy showed no evidence of immune complex deposition. These findings were consistent with AIN with necrotizing glomerulonephritis, suggesting a severe inflammatory response.

Based on the kidney biopsy results, steroid therapy was initiated with intravenous methylprednisolone at a dose of 0.5 mg/kg every 12 h. The patient showed gradual improvement in kidney function over the next week. Hemodialysis was discontinued 7 days after the initiation of steroid therapy, owing to an increase in urine output and a recovery of kidney function. By hospital day 12, the serum creatinine level had decreased to 3.1 mg/dL, the NGAL level had decreased to 315 ng/mL, and the urine output had normalized, indicating improved tubular damage. The steroid dose was tapered over a 4-week period. By the time of discharge, serum creatinine has further improved to 1.4 mg/dL. At the 3-month outpatient follow-up, kidney function remained stable, with a creatinine level of 1.5 mg/dL and no evidence of proteinuria or hematuria on repeat urinalysis.

## 3. Discussion

PT is widely utilized in the treatment of serious bacterial infections due to its broad-spectrum activity and generally favorable safety profile. However, its potential to cause AKI requires careful consideration, particularly in critically ill patients. Although PT-induced AKI is most commonly associated with AIN [5], this case underscores its capacity to induce glomerular injury, such as necrotizing glomerulonephritis, a rare immune-mediated kidney injury that can progress to life-threatening AKI.

PT combines piperacillin, a ureidopenicillin beta-lactam antibiotic, with tazobactam, a beta-lactamase inhibitor, at a fixed ratio of 8:1 [12,13]. This combination has demonstrated robust efficacy in the treatment of poly-microbial infections, including those caused by beta-lactamase-producing bacteria. At steady state, the volume of distribution for piperacillin ranges from 15 to 21 L, whereas that of tazobactam ranges from 18 to 35 L. Both compounds exhibit approximately 20% plasma protein binding. Distribution occurs within 30 min after the end of infusion, and maximum plasma levels are reached within 1 to 2 h. The mean plasma elimination half-life is approximately 0.8 to 1 h; 50–60% of the compound is excreted via the kidneys, and less than 2% is eliminated through biliary excretion. Consequently, dosage adjustments are necessary for patients with renal impairment.

Many studies have investigated the occurrence of AKI associated with PT use, revealing rates of 1.7% to 38.5%. These studies indicated that treatment duration was not associated with AKI incidence. Identified risk factors included advanced age, diabetes, and chronic kidney disease [6,8,11,14,15]. AIN, the most frequently reported PT-related renal complication, is characterized by an immune-mediated hyper-sensitivity reaction that can manifest as a delayed T cell-mediated (type IV) hyper-sensitivity reaction [5]. AIN typically occurs after exposure to a causative agent; it often elicits a more rapid and severe reaction upon re-exposure. The kidneys are particularly susceptible to AIN because they serve as a primary site for the excretion of drugs and their metabolites, which can act as antigens that trigger an immune response [16].

AIN is a form of acute kidney injury characterized by inflammation of the renal interstitium, often triggered by a drug hyper-sensitivity reaction to medications. The condition is often drug-induced, with beta-lactam antibiotics, non-steroidal anti-inflammatory drugs, and proton pump inhibitors among the most common causes [17]. Antibiotics are known to be the most common cause of AIN, and in a case series of 133 patients with biopsy-proven AIN, 70% of the cases were drug-induced, and antibiotics were responsible for almost half of the drug-related AIN [18]. Of the drugs commonly implicated in AIN, beta-lactam antibiotics are the most common causative agents [5]. In the case of beta-lactam antibiotics, including PT, AIN results from an immune-mediated reaction where drug metabolites act as antigens, triggering a cascade of immune responses that lead to kidney injury.

Beta-lactam antibiotics, including piperacillin, are commonly used in both outpatient and inpatient settings to treat various infections and are among the most common antibiotics implicated in AIN. These drugs are eliminated via glomerular filtration and to varying degrees through active transport across tubular epithelial cells [5]. Antigenic determinants form when reactive degradation products of these drugs bind to albumin. The resulting piperacillin-albumin complexes interact with drug-specific T cells, triggering the recruitment of inflammatory cells and eosinophils to the renal interstitium. This immune response leads to inflammation and tubular dysfunction. Activated fibroblasts proliferate and enhance matrix synthesis, eventually causing interstitial fibrosis. Furthermore, infiltrating macrophages release collagenase and reactive oxygen species, exacerbating lymphocyte-initiated injury [5,16]. Notably, kidney injuries in AIN are predominantly confined to the tubules; there is minimal glomerular involvement.

Clinically, AIN typically presents as a non-oliguric AKI approximately 10 days after exposure to the causative agents, with non-specific symptoms, including fever, rash, and eosinophilia, although these classic findings are not always present [19]. Occasionally, eosinophilia and other symptoms of allergic symptoms are seen in AIN, but they are often not present [19,20]. Patients may present with signs of AKI, such as rising serum creatinine levels and electrolyte abnormalities. Urinalysis often reveals sterile pyuria, microscopic hematuria, and eosinophiluria, which may be suggestive but not diagnostic of AIN. A definitive diagnosis is typically made through renal biopsy, which reveals interstitial edema, mononuclear cell infiltration, and varying degrees of tubulitis. In severe cases, fibrosis may develop, leading to chronic kidney dysfunction if not treated promptly. Treatment of AIN primarily involves discontinuation of the offending drug, which often results in partial or complete recovery of kidney function. Steroids are often used in moderate to severe cases, as they can attenuate inflammation and accelerate renal recovery. Studies suggest that early initiation of steroid therapy may improve outcomes, although optimal dosing and duration remain uncertain [21,22]. In cases where kidney dysfunction persists and is severe despite treatment, supportive care, including dialysis, may be required.

Necrotizing glomerulonephritis is the histological hallmark of rapidly progressive glomerulonephritis, which can cause rapid decline in kidney function and a potentially life-threatening condition [23]. It is characterized by segmental areas of fibrinoid necrosis within the glomerular tuft, reflecting severe endothelial injury [23,24]. Although necrotizing glomerulonephritis is rarely associated with antibiotic therapy, cases have been reported after the use of antibiotics such as rifampicin and minocycline [25,26,27,28,29]. Rifampicin is commonly used as one of the first-line drugs in the treatment of tuberculosis. There have been several cases of necrotizing glomerulonephritis after the use of rifampicin, and they usually occurred after 1 or 2 months of initiation of therapy [25,26,27,28]. In most cases, rifampicin was discontinued, and patients responded well to steroids without the use of other treatments such as cyclophosphamide, azathioprine, or plasma exchange. Temporary dialysis was reported in one case, but in the remaining cases, the renal damage was not severe, and the response to the high-dose steroid therapy was good, showing improvement in renal function after treatment. In addition, anti-rifampicin antibodies were detected in some patients with rifampicin-induced necrotizing glomerulonephritis, but there were more cases in which the antibodies were not detected, making it difficult to determine the exact cause of the disease. Sethi et al. reported a necrotizing glomerulonephritis case after minocycline therapy [29]. In that case, circulating cytoplasmic anti-neutrophil cytoplasmic antibodies and anti-proteinase 3 antibodies developed one week after minocycline treatment. Additionally, these antibodies caused a pauci-immune focal necrotizing glomerulonephritis. Fortunately, the patient in this case also responded to discontinuation of minocycline and steroid treatment, and the patient’s renal function improved.

To our knowledge, this is the first reported case of necrotizing glomerulonephritis after PT use. The pathogenesis may involve two mechanisms: (1) direct or indirect activation of glomerular endothelial cells by PT or its metabolites, leading to increased adhesion molecule expression and inflammatory cell recruitment, and (2) overactivation of the complement system, resulting in the formation of circulating immune complexes and triggering immune-mediated glomerulonephritis. Piperacillin is recognized as one of the most immunogenic antibiotics [30]. Piperacillin-induced antibodies have been shown to form immune complexes that predispose patients to autoimmune conditions, such as drug-induced immune hemolytic anemia [31].

Necrotizing glomerulonephritis can cause a rapid decline in kidney function, requiring prompt diagnosis and aggressive treatment. In this case, the patient experienced a rapid improvement in kidney function with the immediate discontinuation of the causative agent and administering high-dose steroid therapy, and no additional immunosuppressive treatment was required. However, in cases of non-response, alternative therapies such as rituximab, cyclophosphamide, and/or plasma exchange should be initiated without delay. We report this case to provide information on the diagnosis, treatment, and clinical course of antibiotic-induced necrotizing glomerulonephritis. Clinicians should remain vigilant for necrotizing glomerulonephritis associated with PT use. Careful monitoring of kidney function and discontinuation of PT or dose reduction in the occurrence of nephrotoxicity may mitigate nephrotoxicity. In cases of rapid deterioration of kidney function, it is necessary to consult with a nephrologist and perform a kidney biopsy.

## 4. Conclusions

This case demonstrates that PT can induce a rare but severe renal complication: AKI with necrotizing glomerulonephritis. Timely kidney biopsy and appropriate immunosuppressive therapy can result in a favorable outcome. Clinicians should be aware of this potential adverse effect and closely monitor kidney function during PT therapy. Additionally, further research is needed to better understand the immunologic mechanisms underlying PT-induced necrotizing glomerulonephritis and to develop strategies for its prevention and treatment.

## Data Availability

The data generated and analyzed in this case are included in the manuscript.
